# Immunological role and prognostic value of SPARCL1 in pan-cancer analysis

**DOI:** 10.3389/pore.2022.1610687

**Published:** 2022-11-22

**Authors:** Kangwei He, Changjiu Li, Hui Yuan, Kang Jiang, Gang Deng

**Affiliations:** ^1^ The Fourth Clinical Medical College, Zhejiang Chinese Medical University, Hangzhou, China; ^2^ Department of Urology Affiliated Hangzhou First People’s Hospital, School of Medicine, Zhejiang University, Hangzhou, China

**Keywords:** prognosis, immune infiltration, pan-cancer, SPARCL1, M2 macrophage

## Abstract

**Background:** Secreted protein acidic and rich in cysteine-like 1 (SPARCL1) was a kind of extracellular matrix glycoprotein. SPARCL1 was strongly inhibited in most cancers. However, the potential functions of SPARCL1 in the pan-cancer cohort have not been widely studied.

**Methods:** We evaluated the transcriptional level and the prognostic value of SPARCL1 in 33 types of cancer and revealed the relationship between genetic alterations of SPARCL1 and the tumor mutation burden. Meanwhile, we assessed the correlations between SPARCL1 and tumor-infiltrating lymphocytes across cancers.

**Results:** The transcriptional level of SPARCL1 was inhibited in most cancers. Although SPARCL1 was down-regulated in most cancers, SPARCL1 might play a protective or detrimental role in different cancers. We demonstrated that mutation count was elevated in the altered SPARCL1 group in several cancers. Additionally, we found a significant positive correlation between SPARCL1 and macrophage infiltration levels in most cancers. Especially, marker sets of M2 macrophages were strongly related to SPARCL1 in cholangiocarcinoma, colon adenocarcinoma, rectum adenocarcinoma, and pancreatic adenocarcinoma.

**Conclusion:** Our study found that SPARCL1 might work as a biomarker for prognosis and immune infiltration in pan-cancer analysis.

## Introduction

Tumorigenesis results from the complex interaction of multiple genes, factors, and signalling pathways, among which misregulation of gene expression plays a critical role. With the implementation of several large genome projects and the development of next-generation sequencing (NGS), a pan-cancer analysis of any potential oncogene is available to explore its mechanisms during initiation, progression, and invasion of cancers.

Secreted protein acidic and rich in cysteine-like 1 (SPARCL1), which belongs to the SPARC family of matricellular proteins, is an extracellular matrix (ECM) glycoprotein [1]. SPARCL1 performs specific functions, including cell adhesion, cell proliferation, and development of the central nervous system by interacting with specific molecules in different ECM environments [2–4]. SPARCL1 was verified to be widely expressed in normal human tissues and acted as an essential role in forming synapses and de-adhesive effects [5–7]. However, the expression level of SPARCL1 was strongly decreased in most human cancers such as colorectal carcinoma, gastric cancer, and prostate carcinoma, suggesting the potential role to reduce cell proliferation and inhibit DNA synthesis [8–10]. The transcriptional level of SPARCL1 was down-regulated in non-small cell lung cancer, supporting the putative function in tumorigenesis [11]. The decreased SPARCL1 in prostate cancer promoted migration of cancer cells [12]. Similar results were obtained in colorectal cancer, which suggested that SPARCL1 inhibited tumor migration and invasion in colorectal cancer and predicted better survival [13]. Unfortunately, the role of SPARCL1 in the pan-cancer cohort remained unclear, and few pan-cancer studies exist to illustrate the relationship between SPARCL1 and multiple tumor types.

We performed a comprehensive analysis of SPARCL1 based on several large genome projects. We assessed the expression level, prognostic value, and genetic alteration of SPARCL1 *via* the TCGA database and GEO database. Moreover, we focused on the association between SPARCL1 transcriptional level and the immune infiltration levels across cancers. This research may be helpful to further comprehend the functions of SPARCL1 and the mechanisms between SPARCL1 and tumor microenvironment.

## Materials and methods

### SPARCL1 expression analysis

We evaluated the transcriptional level of SPARCL1 in different tumors and adjacent normal tissues based on the Gene_DE module of the TIMER2.0 dataset (http://timer.cistrome.org/) [14]. For certain types of cancer not included in the TIMER2.0, GEPIA2 (http://gepia2.cancer-pku.cn/#analysis) was carried out to assess the expression level of SPARCL1 [15]. Briefly, we integrated GTEx and TCGA normal data and evaluated the expression level of SPARCL1 based on the Box Plots module of the GEPIA2 database. The thresholds were verified as the following values: |log2FC| cutoff of 1, *p*-value cutoff of 0.05, and jitter size of 0.4. We then evaluated the relationship between SPARCL1 and the pathological stages of cancers based on the Stage Plot module of the GEPIA2 database.

### Prognostic value analysis

To observe the prognostic value of SPARCL1, survival analysis was carried out to evaluate the overall survival (OS) and the disease-free survival (DFS) between the high SPARCL1 expression group and the low SPARCL1 expression group *via* the Survival Map module of GEPIA2. Median was used as the cutoff value for grouping. We then evaluated the OS of different expression level of SPARCL1 in breast (*n* = 1402), ovarian (*n* = 1656), lung (*n* = 1925), and gastric (*n* = 875) cancer based on Kaplan-Meier plotter (http://kmplot.com/analysis/) [16]. Median was used as the cutoff value for grouping. In the PrognoScan database (http://dna00.bio.kyutech.ac.jp/PrognoScan/index.html), we evaluated the association between SPARCL1 transcriptional level and the prognostic value, such as the OS and the relapse-free survival (RFS), in several cancers, including bladder, blood, breast, colorectal, lung, ovarian, and prostate cancer [17]. The optimal cutpoint was used as the cutoff value for grouping.

### Genetic alterations analysis

The pan-cancer TCGA cohort (*n* = 10,950 from 32 kinds of cancers) was obtained from the cBioPortal database (https://www.cbioportal.org/) [18]. We analyzed the genetic alterations (mutation, fusion, amplification, and deep deletion) of SPARCL1 in the TCGA cohort based on the Cancer Types Summary module of cBioPortal. Besides, the Comparison/Survival module was performed to explore the association between SPARCL1 and clinical features of the pan-cancer cohort.

### Functional enrichment analysis

We found other genes that are related to SPARCL1 by STRING and obtained the Entrez Gene ID by “org.Hs.eg.db” R package. We carried out Kyoto Encyclopedia of Genes and Genomes (KEGG) pathway enrichment analysis using the “clusterProfiler” R package and visualized the results by “enrichplot” and “ggplot2” R packages.

### Immune infiltration analysis

The relationship between SPARCL1 transcriptional level and the infiltrates level of six immune cell types (B cells, CD4^+^ T cells, CD8^+^ T cells, neutrophils, macrophages, and dendritic cells) in pan-cancer was acquired from TIMER database (https://cistrome.shinyapps.io/timer/) [19]. Besides, the correlation between SPARCL1 transcriptional level and immune markers of immune cell types was acquired from TIMER database and the scatterplots was drawn by the correlation module [19].

### Statistical analysis

The transcriptional level of SPARCL1 was compared based on the Wilcoxon test, whereas the log-rank test was carried out for Kaplan-Meier analysis. Spearman’s rank correlation test was carried out to evaluate the correlation of SPARCL1 expression level and infiltrates levels and immune marker sets. The cor value greater than 0.4 indicates a moderate correlation, the cor value greater than 0.6 indicates a strong correlation, and the cor value greater than 0.8 indicates a very strong correlation. All statistical analyses were performed using IBM SPSS Statistics 22.0, and R software 4.0.0. A value of *p* < 0.05 was considered statistically significant for all statistical analyses.

## Results

### Aberrant expression of SPARCL1 in TCGA across cancers

**FIGURE 1:**
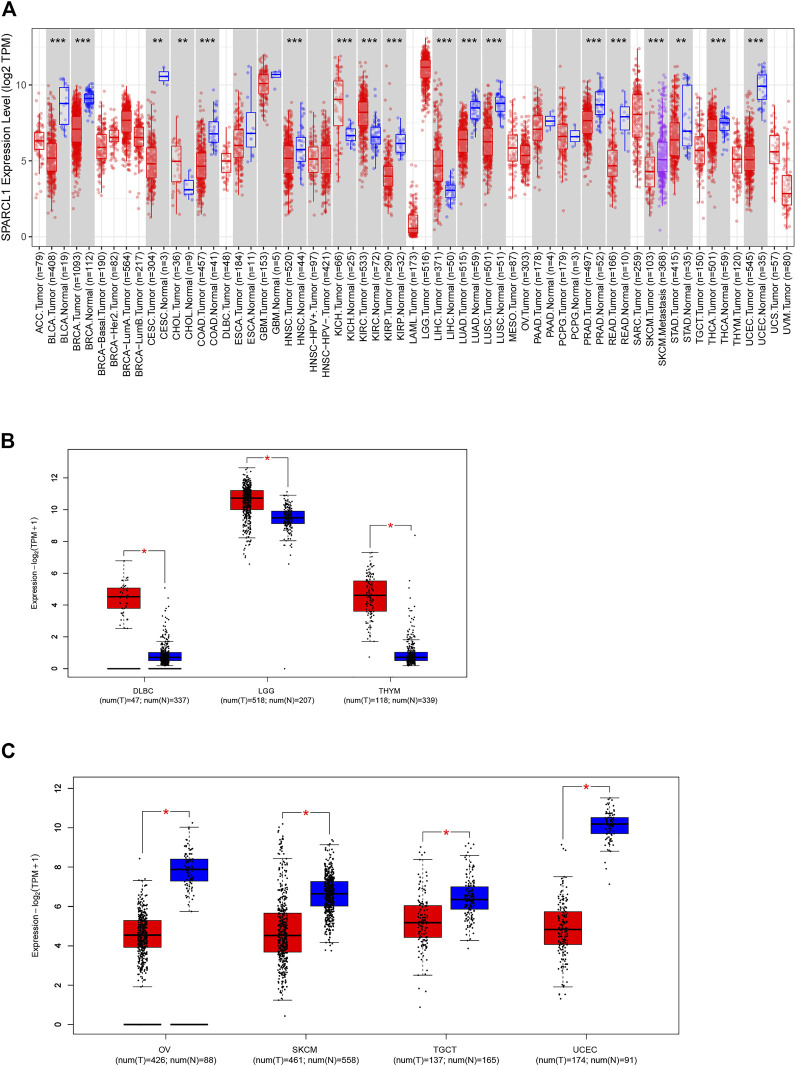
Aberrant expression of SPARCL1 in TCGA across cancers. **(A)** The expression level of SPARCL1 in different cancers. * indicates *p* < 0.05, ** indicates *p* < 0.01, *** indicates *p* < 0.001. **(B)** The expression level of SPARCL1 was increased in DLBC, LGG, and THYM. * indicates *p* < 0.001. **(C)** The expression level of SPARCL1 was decreased in OV, SKCM, TGCT, and UCEC. * indicates *p* < 0.001.

As previous results demonstrated that SPARCL1 was down-regulated in several human tumors, we identified the expression of SPARCL1 in TCGA across cancers. The expression of SPARCL1 in different tumor tissues and adjacent normal tissues was retrieved from the TIMER2.0 database. By comparing the SPARCL1 transcriptional level in tumor and normal tissues, we demonstrated that SPARCL1 was generally aberrantly expressed in most tumor tissues compared with matched normal tissues. The SPARCL1 expression level was elevated in kidney chromophobe (KICH), kidney renal clear cell carcinoma (KIRC), liver hepatocellular carcinoma (LIHC) (*p* < 0.001), and cholangiocarcinoma (CHOL) (*p* < 0.01) (Figure 1A). Contrarily, SPARCL1 was decreased in breast invasive carcinoma (BRCA), bladder urothelial carcinoma (BLCA), colon adenocarcinoma (COAD), head and neck squamous cell carcinoma (HNSC), kidney renal papillary cell carcinoma (KIRP), lung adenocarcinoma (LUAD), lung squamous cell carcinoma (LUSC), prostate adenocarcinoma (PRAD), rectum adenocarcinoma (READ), thyroid carcinoma (THCA), uterine corpus endometrial carcinoma (UCEC) (*p* < 0.001), stomach adenocarcinoma (STAD), and cervical endocervical cancer (CESC) (*p* < 0.01).

We next integrated the GTEx database and TCGA database and assessed the SPARCL1 transcriptional level in 11 other cancers, including adrenocortical carcinoma (ACC), lymphoid neoplasm diffuse large B-cell lymphoma (DLBC), acute myeloid leukemia (LAML), brain lower-grade glioma (LGG), ovarian serous cystadenocarcinoma (OV), sarcoma (SARC), skin cutaneous melanoma (SKCM), testicular germ cell tumors (TGCT), thymoma (THYM), uterine carcinosarcoma (UCS), and uveal melanoma (UVM). SPARCL1 had been found to have increased expression in DLBC, LGG, and THYM (*p* < 0.001) and have reduced expression in OV, SKCM, TGCT, and UCEC (*p* < 0.001) (Figures 1B,C). In contrast, no statistical difference was evident in ACC, LAML, and SARC compared with normal tissues (Supplementary Figure S1).

**FIGURE 2:**
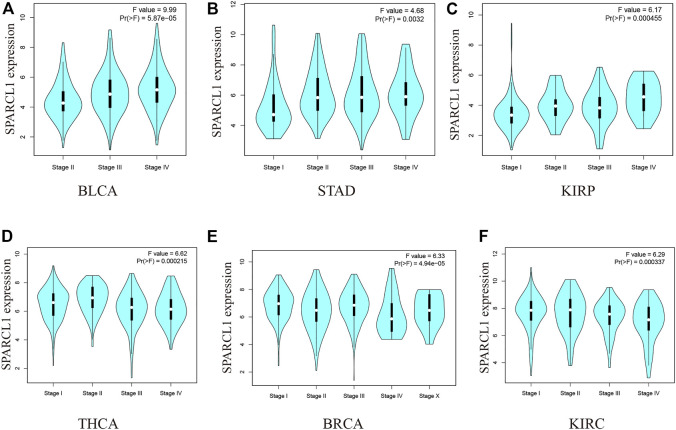
**(A–C)** The expression level of SPARCL1 elevated as the pathological stages of BLCA, KIRP, and STAD increased. **(D–F)** The expression level of SPARCL1 decreased as the pathological stages of BRCA, KIRC, and THCA increased.

We then evaluated the association between SPARCL1 and the pathological stages across cancer types. As shown in Figures 2A–F and Supplementary Figure S2, the expression level of SPARCL1 was elevated as the pathological stages of BLCA, KIRP, and STAD increased while low expression of SPARCL1 increased the likelihood of low pathological stages in BRCA, KIRC, and THCA.

### Prognostic value of SPARCL1 across cancer types

**FIGURE 3:**
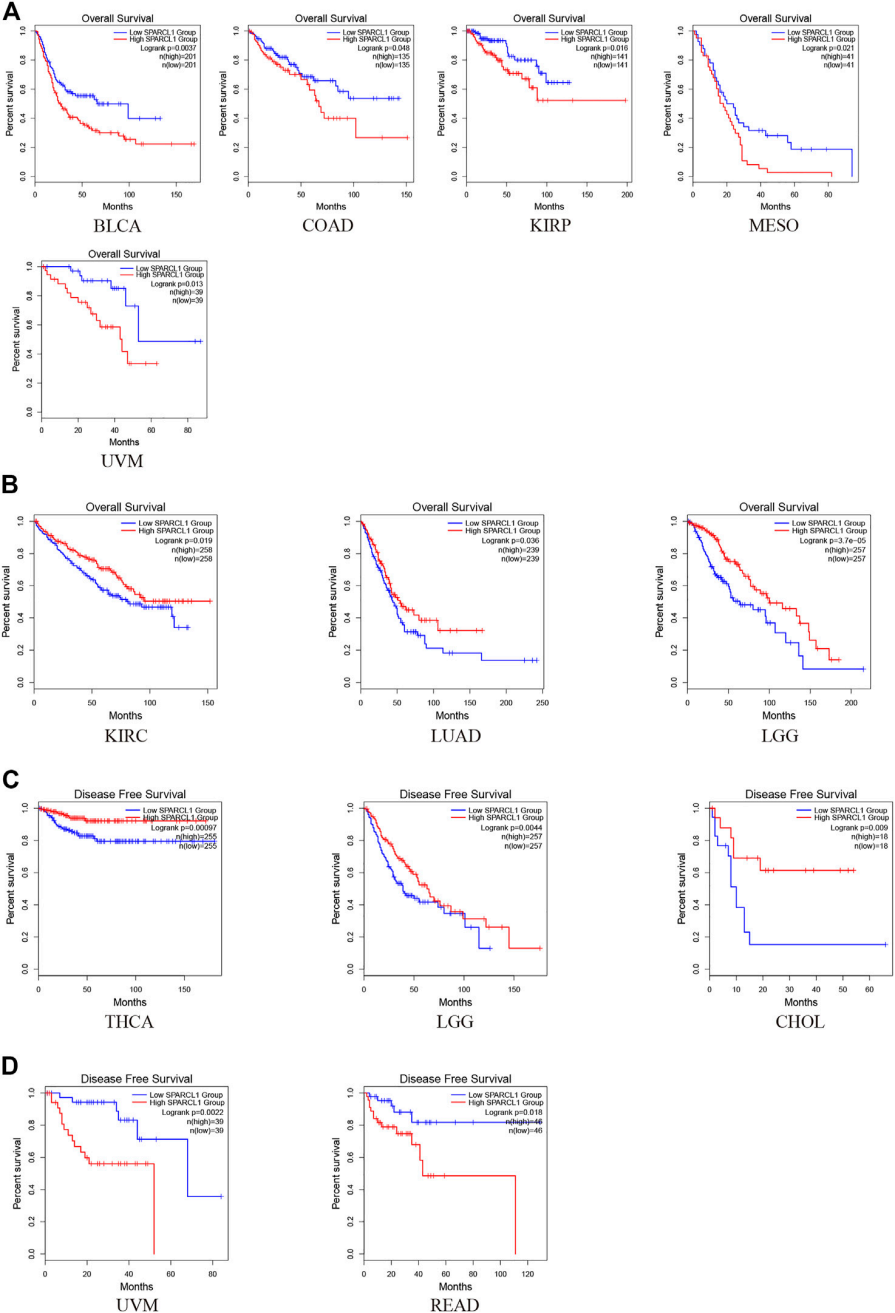
The prognostic value of SPARCL1 across cancer types based on GEPIA2 database. **(A)** Patients with high SPARCL1 expression level had a worse OS than those with low SPARCL1 level in BLCA, COAD, KIRP, MESO, and UVM. **(B)** Patients with high SPARCL1 expression level had a better OS than those with low SPARCL1 level in KIRC, LGG, and LUAD. **(C)** Low expression of SPARCL1 was linked with a poor DFS for CHOL, LGG, and THCA. **(D)** High expression of SPARCL1 was related to a poor DFS in READ and UVM.

To investigate the impact of SPARCL1 across cancer types, normalized RNA-seq data were utilized to compare the prognosis in different SPARCL1 expression levels in 33 types of cancer *via* GEPIA2. Remarkably, we revealed that SPARCL1 expression level impacted the OS in 8 types of cancers, including BLCA, COAD, KIRP, MESO, UVM, KIRC, LGG, and LUAD. The up-regulated SPARCL1 was linked with a poor OS in BLCA, COAD, KIRP, MESO, and UVM (Figure 3A). Contrarily, patients with low SPARCL1 expression had a reduced OS in KIRC, LGG, and LUAD (Figure 3B). Then the disease-free survival (DFS) analysis was performed to further assess the prognostic value of SPARCL1 across cancers. The decreased SPARCL1 was linked with a poor DFS in CHOL, LGG, and THCA (Figure 3C). However, as shown in Figure 3D, high SPARCL1 expression was associated with a worse DFS in READ and UVM.

**FIGURE 4:**
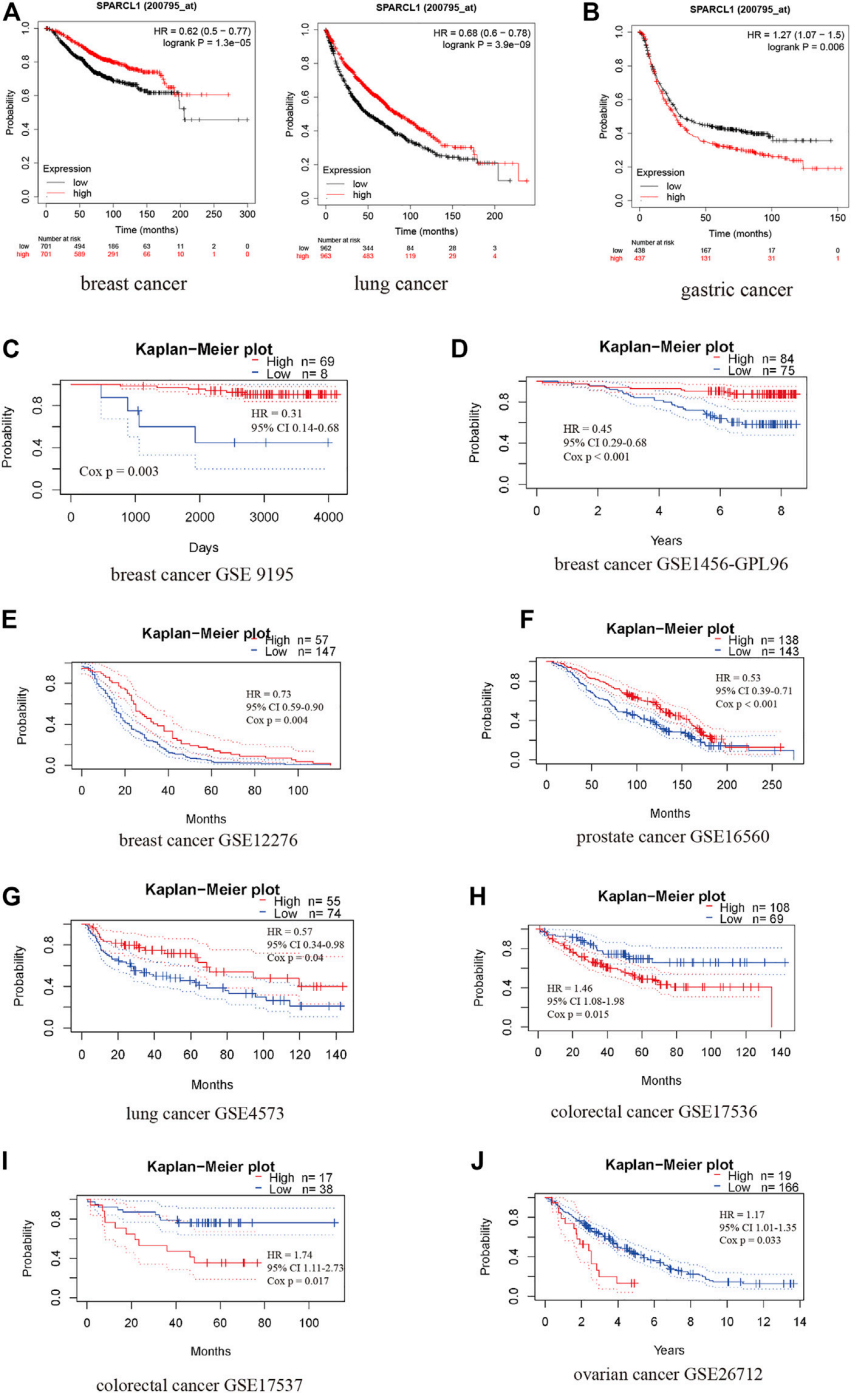
The prognostic value of SPARCL1 across cancer types based on Kaplan-Meier plotter database **(A,B)** and PrognoScan database **(C–J)**. **(A)** High expression of SPARCL1 was related to a good prognosis in lung cancer and breast cancer. **(B)** High expression of SPARCL1 was related to a poor prognosis in gastric cancer. **(C–E)** High expression of SPARCL1 was related to a good prognosis in breast cancer. **(F,G)** High expression of SPARCL1 was related to a good prognosis in prostate cancer and lung cancer. **(H–J)** High expression of SPARCL1 was related to a poor prognosis in colorectal cancer and ovarian cancer.

In addition to survival analysis based on RNA-seq data, survival analysis based on microarray data was performed to further assess the prognostic value of SPARCL1. Intriguingly, highly expressed SPARCL1 was related to good prognosis in lung and breast cancer based on Kaplan-Meier plotter database (HR = 0.68, *p* = 3.9e-09 and HR = 0.62, *p* = 1.3e-05, respectively) (Figure 4A). In contrast, SPARCL1 expression had negative effects on the prognosis in gastric cancer (HR = 1.27, *p* = 0.006) (Figure 4B). PrognoScan data suggested that SPARCL1 expression level might influence the survival time in some breast, colorectal, ovarian, prostate, and lung cancer cohorts (Supplementary Table S1). Three breast cancer cohorts demonstrated that patients with increased SPARCL1 have a better prognosis (GSE 9195, RFS HR = 0.31, Cox *p* = 0.003; GSE1456-GPL96, OS HR = 0.45, Cox *p* < 0.001; GSE12276, RFS HR = 0.73, Cox *p* = 0.004) (Figures 4C–E). Additionally, the good prognosis was verified to be associated with elevated SPARCL1 level in a prostate cancer cohort (GSE16560, OS HR = 0.53, Cox *p* < 0.001) and a lung cancer cohort (GSE4573, OS HR = 0.57, Cox *p* = 0.04) and decreased SPARCL1 level in a colorectal cancer cohort (GSE17536, OS HR = 1.46, Cox *p* = 0.015; GSE17537, OS HR = 1.74, Cox *p* = 0.017) and an ovarian cancer cohort (GSE26712, OS HR = 1.17, Cox *p* = 0.033) (Figures 4F–J).

As described, these results supported prognostic implications of SPARCL1 in pan-cancer. Although these results provided a broad view of the prognosis across cancers, more underlying mechanisms studies and larger sample size studies are warranted.

### Genetic alterations of SPARCL1 and the relationship with TMB

**FIGURE 5:**
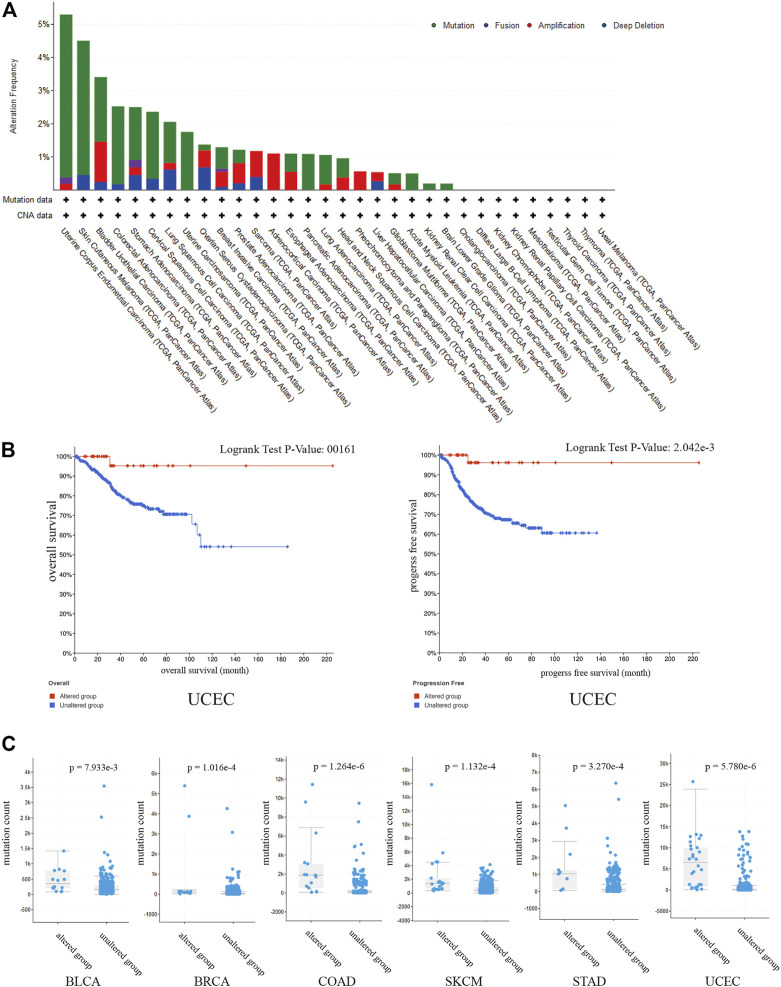
**(A)** The alteration frequency and types of the SPARCL1 in 32 type cancers. **(B)** UCEC patients with altered SPARCL1 had a better OS and RFS than those with unaltered SPARCL1. **(C)** The mutation count was elevated in altered SPARCL1 group compared with unaltered SPARCL1 group in UCEC, SKCM, BLCA, COAD, STAD, and BRCA.

To evaluate the prevalence of SPARCL1 genetic alterations, we queried the pan-cancer TCGA atlas (*n* = 10,950). Figure 5A showed the alteration frequency and types of the SPARCL1 gene in 32 types of cancer. Overall, 1.5% (162/10,950) of all cancers possessed SPARCL1 genetic alteration status. The highest alteration frequency of SPARCL1 appeared in UCEC (>5%), followed by SKCM and BLCA (>3%). We also observed that UCEC patients with altered SPARCL1 had a better OS (*p* = 0.016) and RFS (*p* = 0.002) than those with unaltered SPARCL1, but not DFS (*p* = 0.178) and disease-specific survival (DSS) (*p* = 0.055) (Figure 5B and Supplementary Figure S3). Additionally, we demonstrated that mutation count was elevated in the altered SPARCL1 group in UCEC, SKCM, BLCA, COAD, STAD, and BRCA (*p* < 0.05) (Figure 5C). Tumor mutation burden (TMB) has been described as an effective biomarker to predict the efficacy of immunotherapeutic response [20]. These results indicated the potential value of SPARCL1 genetic alterations in evaluating immunotherapeutic response.

### Potential functions of SPARCL1 across cancers

**FIGURE 6:**
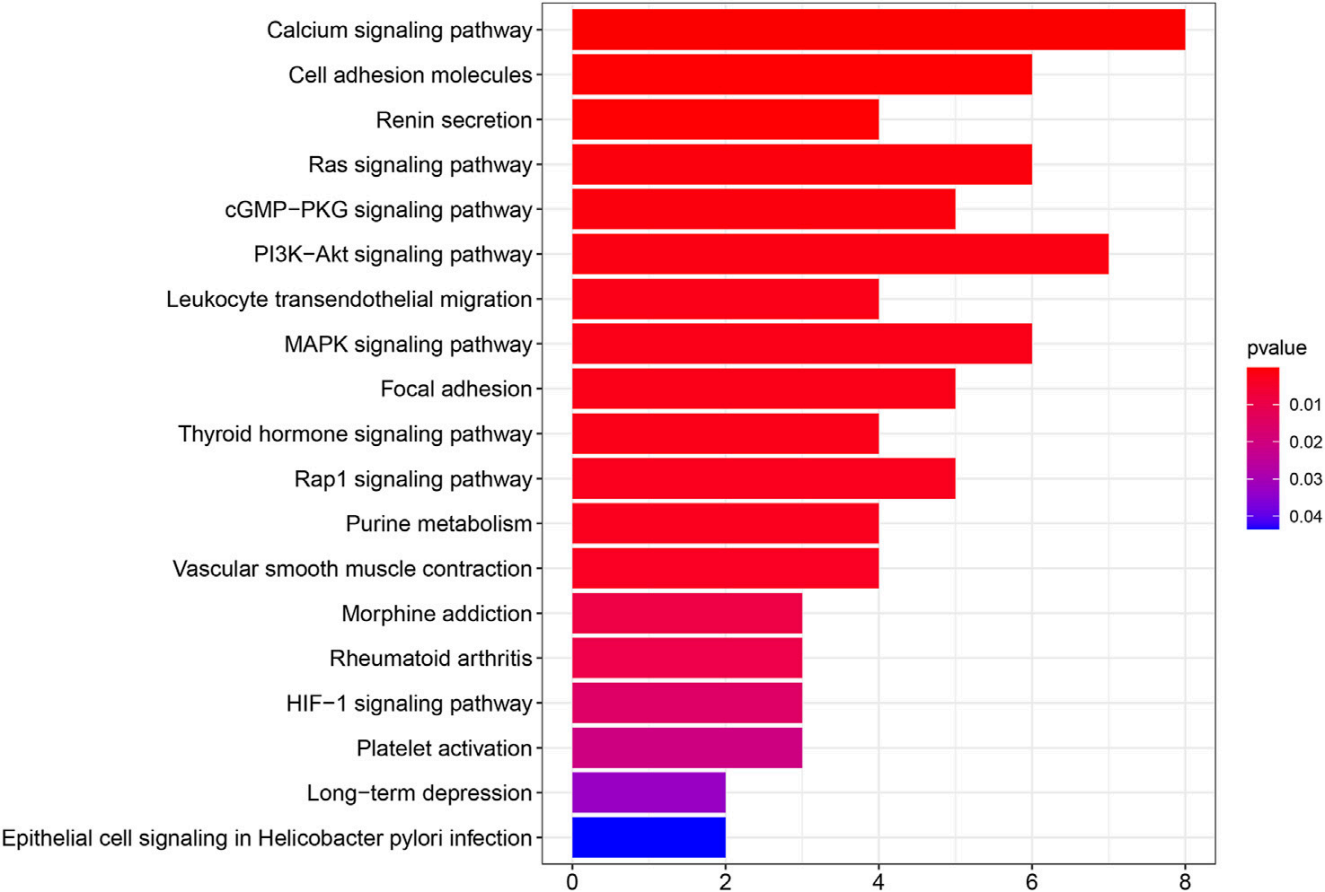
The results of KEGG pathway enrichment analysis.

We performed PPI network analysis to evaluate genes that are related to SPARCL1 (Supplementary Table S2). Then we conducted KEGG pathway enrichment analysis to evaluate the potential functions of SPARCL1 and neighboring genes. As shown in Figure 6, in the KEGG pathway enrichment analysis, SPARCL1 and related genes were highly enriched in calcium signaling pathway, PI3K-Akt signaling pathway, Cell adhesion molecules, Ras signaling pathway, MAPK signaling pathway, cGMP-PKG signaling pathway.

### Immune infiltration level of SPARCL1 across cancers

**FIGURE 7:**
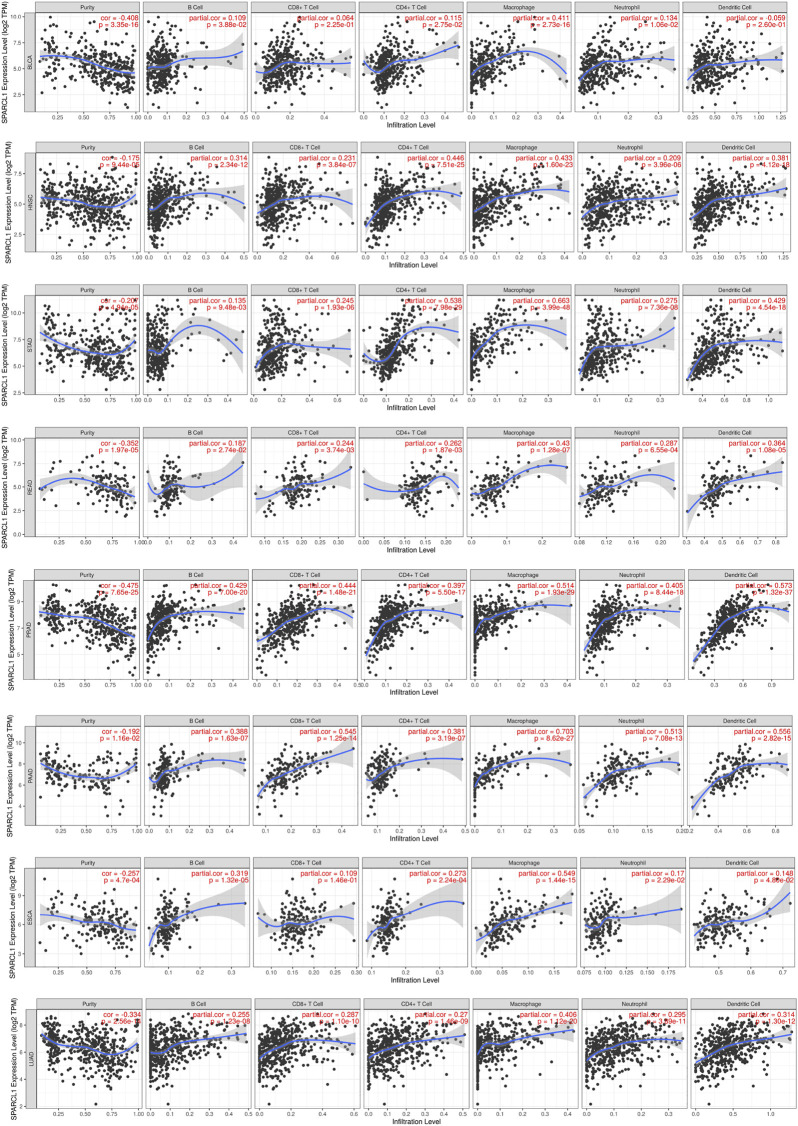
Correlation of SPARCL1 with immune infiltration level in BLCA, HNSC, STAD, READ, PRAD, PAAD, ESCA, and LUAD.

Although previous results indicated the potential prognostic value of SPARCL1 across cancer, its potential role is still unknown. Tumor-infiltrating lymphocytes of the local microenvironment of tumor have been proven either tumor-suppressive or tumor-promoting functions [21]. However, whether SPARCL1 was associated with immune infiltration level remained unclear. We evaluated the correlation between SPARCL1 transcriptional level and tumor-infiltrating lymphocytes across cancers. The score of six kinds of immune cells, including B cells, CD4^+^ T cells, CD8^+^ T cells, neutrophils, macrophages, and dendritic cells, were obtained from the TIMER database. We showed a remarkable positive association between SPARCL1 and macrophages infiltration level in BLCA, BRCA, CESC, ESCA, GBM, HNSC, KIRC, LUAD, LUSC, OV, PAAD, PRAD, READ, SKCM, STAD, TGCT, UCEC, and UCS. Additionally, elevated SPARCL1 also had correlations with elevated infiltration level of all kinds of immune cells in BRCA, CESC, HNSC, LUAD, LUSC, PAAD, PRAD, READ, and STAD. Contrarily, no correlation was evident between SPARCL1 and tumor-infiltrating lymphocytes level in DLBC, LIHC, LGG, and UVM. The details were displayed in (Figure 7 and Supplementary Figures S4–S6). These results indicated that SPARCL1 was associated with immune infiltration across cancers.

### Correlation analysis between SPARCL1 and immune markers

Previous results revealed a significant association between SPARCL1 and tumor-infiltrating lymphocytes in most cancers, we sought to verify the correlation between SPARCL1 and immune marker sets from the TIMER database. A correlation was observed between CCL2, CD68, and IL10 of macrophages and SPARCL1 expression in BLCA, BRCA, CESC, COAD, DLBC, HNSC, LUAD, LUSC, OV, PAAD, PCPG, PRAD, STAD, THYM, UCEC, UVM, and READ (Table 1 and Supplementary Table S3). We revealed that CD8A and CD8B of CD8^+^ T cells were remarkably associated with SPARCL1 expression in BLCA, BRCA, CESC, COAD, ESCA, HNSC, LUAD, LUSC, OV, MESO, PAAD, PRAD, STAD, SKCM, KIRC, KIRP, THYM, UCEC, and UVM. Besides, a significant correlation was confirmed between CD86 and CD115 of monocyte and SPARCL1 in BLCA, BRCA, CESC, COAD, ESCA, HNSC, LUAD, LUSC, OV, PAAD, PRAD, STAD, THYM, UCEC, UVM, READ, UCS, SARC, SKCM, and KIRP. Especially, a strong correlation was observed between SPARCL1 and CCL2 of macrophages in COAD and READ (cor = 0.711 and 0.732, respectively), CD115 of monocyte in COAD, PAAD, PRAD, and THYM (cor = 0.615, 0.72, 0.613, and 0.63, respectively), CD86 of monocyte in PAAD and READ (cor = 0.62 and 0.656, respectively), and CD8A of CD8^+^ T cells in PAAD (cor = 0.695). We further verified the association between SPARCL1 and immune marker sets of M1 and M2 macrophages. SPARCL1 expression had relationships with the infiltration level of M1 macrophages and M2 macrophages (Table 2 and Supplementary Table S4). Especially, CD163, VSIG4, and MS4A4A of M2 macrophages were strongly correlated with SPARCL1 expression in CHOL, COAD, PAAD, and READ. As described, these findings further elucidated that SPARCL1 was possibly associated with the macrophages infiltration, especially M2 macrophages.

**TABLE 1:** Correlation analysis between SPARCL1 and immune marker sets of CD8^+^ T cells, monocyte and macrophage.

Description	Markers	BLCA		BRCA		CESC		COAD		DLBC		ESCA	
		Cor	P	Cor	P	Cor	P	Cor	P	Cor	P	Cor	P
CD8^+^ T cell	CD8A	0.242	***	0.293	***	0.286	***	0.362	***	0.409	**	0.381	***
	CD8B	0.179	***	0.197	***	0.243	***	0.253	***	0.208	0.16	0.338	***
Monocyte	CD86	0.369	***	0.173	***	0.326	***	0.64	***	0.249	0.09	0.474	***
	CD115	0.475	***	0.341	***	0.402	***	**0.615**	***	0.44	**	0.57	***
Macrophage	CCL2	0.505	***	0.172	***	0.4	***	**0.711**	***	0.404	**	0.556	***
	CD68	0.245	***	0.146	***	0.134	*	0.49	***	0.375	**	0.143	0.05
	IL10	0.496	***	0.181	***	0.305	***	0.496	***	0.483	***	0.42	***

		**HNSC**		**LUAD**		**LUSC**		**OV**		**PAAD**		**PCPG**	
		**Cor**	**P**	**Cor**	**P**	**Cor**	**P**	**Cor**	**P**	**Cor**	**P**	**Cor**	**P**
CD8^+^ T cell	CD8A	0.224	***	0.222	***	0.24	***	0.24	***	**0.695**	***	-0.037	0.63
	CD8B	0.219	***	0.149	***	0.156	***	0.234	***	0.566	***	0.166	*
Monocyte	CD86	0.405	***	0.373	***	0.256	***	0.321	***	**0.62**	***	0.141	0.06
	CD115	0.476	***	0.429	***	0.335	***	0.405	***	**0.72**	***	0.202	**
Macrophage	CCL2	0.535	***	0.265	***	0.281	***	0.153	0.00766	0.522	***	0.21	**
	CD68	0.305	***	0.346	***	0.267	***	0.337	***	0.36	***	0.268	***
	IL10	0.475	***	0.384	***	0.205	***	0.382	***	0.599	***	0.233	**

		**PRAD**		**STAD**		**THYM**		**UCEC**		**UVM**		**READ**	
		**Cor**	**P**	**Cor**	**P**	**Cor**	**P**	**Cor**	**P**	**Cor**	**P**	**Cor**	**P**
CD8^+^ T cell	CD8A	0.422	***	0.318	***	-0.294	**	0.311	***	0.536	***	0.302	***
	CD8B	0.196	***	0.177	***	-0.403	***	0.109	0.0112	0.514	***	0.145	0.06
Monocyte	CD86	0.499	***	0.374	***	0.56	***	0.323	***	0.541	***	**0.656**	***
	CD115	**0.613**	***	0.508	***	**0.63**	***	0.267	***	0.417	***	0.525	***
Macrophage	CCL2	0.31	***	0.512	***	0.478	***	0.194	***	0.466	***	**0.732**	***
	CD68	0.414	***	0.205	***	0.403	***	0.156	***	0.255	*	0.392	***
	IL10	0.385	***	0.39	***	0.259	**	0.133	**	0.498	***	0.335	***

* indicates *p* < 0.05, ** indicates *p* < 0.01, *** indicates *p* < 0.001. Bold value indicates cor > 0.6.

**TABLE 2:** Correlation analysis between SPARCL1 and immune marker sets of M1 and M2 macrophage.

		BLCA		STAD		TGCT		CHOL		COAD		UVM	
		cor	p	cor	p	cor	p	cor	p	cor	p	cor	p
M1 Macrophage	INOS	0.211	***	−0.037	0.45	0.441	***	0.22	0.20	−0.187	***	0.192	0.09
	IRF5	0.102	*	0.232	***	0.333	***	0.337	*	0.298	***	0.508	***
	COX2	0.235	***	0.265	***	0.443	***	0.466	**	0.325	***	**0.644**	***
M2 Macrophage	CD163	0.474	***	0.431	***	0.361	***	**0.608**	***	**0.66**	***	0.534	***
	VSIG4	0.439	***	0.424	***	0.273	***	0.403	*	**0.614**	***	0.442	***
	MS4A4A	0.47	***	0.492	***	0.338	***	0.56	***	**0.614**	***	0.495	***

		**DLBC**		**ESCA**		**HNSC**		**LUAD**		**LUSC**		**OV**	
		**cor**	**p**	**cor**	**p**	**cor**	**p**	**cor**	**p**	**cor**	**p**	**cor**	**p**
M1 Macrophage	INOS	0.431	**	0.063	0.40	0.343	***	0.373	***	0.114	*	0.268	***
	IRF5	0.317	*	0.115	0.12	0.17	***	0.163	***	0.254	***	0.151	**
	COX2	0.472	***	0.182	*	0.067	0.13	0.111	*	−0.057	0.2	0.242	***
M2 Macrophage	CD163	0.439	**	0.506	***	0.495	***	0.41	***	0.372	***	0.421	***
	VSIG4	0.333	*	0.494	***	0.448	***	0.327	***	0.341	***	0.378	***
	MS4A4A	0.56	***	0.536	***	0.472	***	0.432	***	0.359	***	0.421	***

		**PAAD**		**PCPG**		**PRAD**		**READ**		**THYM**		**UCEC**	
		**cor**	**p**	**cor**	**p**	**cor**	**p**	**cor**	**p**	**cor**	**p**	**cor**	**p**
M1 Macrophage	INOS	0.223	**	−0.146	0.0504	0.239	***	−0.254	**	0.436	***	0.095	*
	IRF5	0.276	***	0.149	0.046	0.236	***	0.151	0.05	0.452	***	0.062	0.15
	COX2	0.138	0.07	0.312	***	0.346	***	0.32	***	**0.618**	***	0.126	**
M2 Macrophage	CD163	**0.69**	***	0.389	***	0.474	***	**0.632**	***	0.526	***	0.251	***
	VSIG4	0.579	***	0.3	***	0.528	***	0.521	***	0.572	***	0.222	***
	MS4A4A	**0.687**	***	0.412	***	0.498	***	**0.632**	***	0.427	***	0.347	***

* indicates *p* < 0.05, ** indicates *p* < 0.01, *** indicates *p* < 0.001. Bold value indicates cor > 0.6.

## Discussion

SPARCL1 is an ECM glycoprotein and functions in cell adhesion and development of the central nervous system [2–4]. Previous research has reported that down-regulated SPARCL1 occurred frequently in most human cancers and emerging publications have reported the potential role of SPARCL1 to reduce cell proliferation and inhibit DNA synthesis [8–10]. Nevertheless, the role of SPARCL1 across cancers has not yet been widely elucidated and few pan-cancer studies exist to suggest the relationship between SPARCL1 and various tumor types.

In this manuscript, we assessed the expression of SPARCL1 across cancers in the TIMER2.0 and GEPIA2 databases. We revealed the transcriptional level of SPARCL1 was down-regulated in most tumor types, including in BRCA, BLCA, COAD, HNSC, KIRP, LUAD, LUSC, PRAD, READ, THCA, UCEC, CESC, STAD, OV, SKCM, TGCT, and UCEC, but elevated in KICH, KIRC, LIHC, CHOL, DLBC, LGG, and THYM. This result suggested SPARCL1 might function as an essential tumor suppressor gene across cancers, which was similar to previous studies [1, 22]. SPARCL1 was significantly downregulated and was associated with tumor stage, distant metastasis, and OS in colorectal cancer [23]. Although most studies verified that SPARCL1 was a potential tumor suppressor gene across cancer types [22, 24, 25], the results were not completely consistent, indicating different biological mechanisms of SPARCL1 in different cancers. SPARCL1 was mostly undetectable in normal liver tissues but was verified up-regulated in hepatocellular carcinoma (HCC) [26, 27]. Intriguingly, overexpression of SPARCL1 in HCC cells remarkably inhibited tumor growth *in vivo* [26]. It is commonsensible that cancer is a highly heterogeneous disease, and the crosstalk within the tumor is diverse. The function of the same gene might be different in different cancers and the function of the same gene in a particular cancer might also be diverse. We supposed that SPARCL1 played different roles that can promote tumorigenesis and inhibit tumor growth in different ECM environment. More researches are needed to explore the potential mechanism of SPARCL1 across cancers.

To explore the prognostic value of SPARCL1 across cancers, the survival analysis was performed with data across databases. Analysis of SPARCL1 in the GEPIA2 database demonstrated that patients with down-regulated SPARCL1 were associated with favorable prognosis in BLCA, COAD, KIRP, MESO, and UVM. Contrarily, reduced SPARCL1 was related to poor prognosis in KIRC, LGG, and LUAD. Analysis of microarray data revealed that up-regulated SPARCL1 was linked with a good prognosis in lung and breast cancer. In contrast, SPARCL1 expression had negative effects on the prognosis in gastric cancer. PrognoScan data suggested that the good prognosis was verified to be associated with elevated SPARCL1 in a breast cohort, a prostate cohort, and a lung cancer cohort and decreased SPARCL1 in a colorectal cohort and an ovarian cancer cohort. These survival analysis results were not completely consistent across databases. For lung cancer, analysis of microarray data found a correlation between elevated SPARCL1 and a good survival time. Additionally, analysis of the TCGA database indicated a correlation between elevated SPARCL1 and a good survival time of LUAD but not LUSC. Nevertheless, some studies demonstrated that SPARCL1 expression had less influence on some lung adenocarcinoma cohorts (GSE 31210 and GSE 13213) [28, 29]. Furthermore, different survival analyses for the SPARCL1 gene demonstrated distinct conclusions for the same tumor. A previous study found poor survival time of gastric cancer patients was linked with lower SPARCL1 transcriptional level [10], which was opposite to our analysis. Although these results provided a broad view of the prognosis across cancers, more underlying mechanisms studies and larger sample size studies are still warranted.

We also demonstrated that mutation count was elevated in the altered SPARCL1 group in UCEC, SKCM, BLCA, COAD, STAD, and BRCA. As TMB has emerged as a reliable predictor of immunotherapy response in many cancers [30], SPARCL1 might function as a biomarker in evaluating immunotherapy response.

Another major finding was the correlation of SPARCL1 and diverse immune infiltration levels across cancers. SPARCL1 was positively associated with macrophages infiltration level in BLCA, BRCA, CESC, ESCA, GBM, HNSC, KIRC, LUAD, LUSC, OV, PAAD, PRAD, READ, SKCM, STAD, TGCT, UCEC, and UCS. Besides, positive correlations were observed between SPARCL1 expression and the infiltration levels of five other immune cells, including B cells, CD4^+^ T cells, CD8^+^ T cells, neutrophils, and dendritic cells. These findings indicated the possible relation between SPARCL1 and immune infiltration cells. Moreover, the associations between SPARCL1 and marker sets of macrophages, CD8^+^ T cells, and monocyte were verified. Especially, immune marker sets of M2 macrophages demonstrated moderate or strong correlations with SPARCL1 across cancers.

Persuasive evidence revealed the considerable functions of macrophages in cancer initiation and malignant progression, including angiogenesis, invasion, and intravasation [31, 32]. However, few studies focused on the correlations between SPARCL1 and macrophages. A previous study verified the potential mechanism which explained why SPARCL1 can recruit macrophages in osteosarcoma [25]. SPARCL1 can activate WNT/β-catenin signaling which recruited macrophages *via* increasing CCL5 secretion [25]. Yet, the functional importance of SPARCL1 in epithelial carcinoma is still unclear, as does the importance of their interactions with macrophages.

Macrophages demonstrated extreme heterogeneity in the tumor microenvironment. Macrophages in tumor microenvironment are polarized into M1 or M2 macrophages in accordance with the external stimulus [33]. The potential function of these two types of macrophages is almost opposed to one another. M2 macrophages demonstrated correlations with tumor progression and poor prognosis while M1 macrophages were associated with anti-metastatic effects and good prognosis [32, 34, 35]. Our findings suggested that SPARCL1 expression was associated with M2 macrophages infiltration level. These results might explain why elevated SPARCL1 expression indicated a poor prognosis in substantial types of tumors.

This study has several limitations. We found SPARCL1 acted as an important tumor suppressor gene during tumorigenesis. Further research would be performed to detect the underlying mechanism. Besides, although SPARCL1 was a potential tumor suppressor gene, survival analysis did not find prominent correlations between high SPARCL1 expression with good prognosis across cancers. As M2 macrophages demonstrated moderate and strong correlations with SPARCL1 expression across cancers, it would be interesting to study the underlying mechanism between SPARCL1 and the M2 macrophages.

In summary, we demonstrated that SPARCL1 was down-regulated in most cancer types and correlated with the pathological stages and the prognosis across cancer types. We further found mutation count was elevated in the altered SPARCL1 group. Additionally, SPARCL1 was associated with M2 macrophages infiltration.

## Data Availability

The data of this study are available from the TIMER2.0 database (http://timer.cistrome.org/), the GEPIA2 database (http://gepia2.cancer-pku.cn/#analysis), the Kaplan-Meier plotter database (http://kmplot.com/analysis/), the PrognoScan database (http://dna00.bio.kyutech.ac.jp/PrognoScan/index.html), the cBioPortal database (https://www.cbioportal.org/), and the TIMER database (https://cistrome.shinyapps.io/timer/). The used codes were deposited in Supplement Material.
