# Supplementary figures and images for: Immunological role and prognostic value of SPARCL1 in pan-cancer analysis
Source: Pathol Oncol Res. 2022 Nov 22;28:1610687. doi: 10.3389/pore.2022.1610687 (PMC9722748; doi:10.3389/pore.2022.1610687)

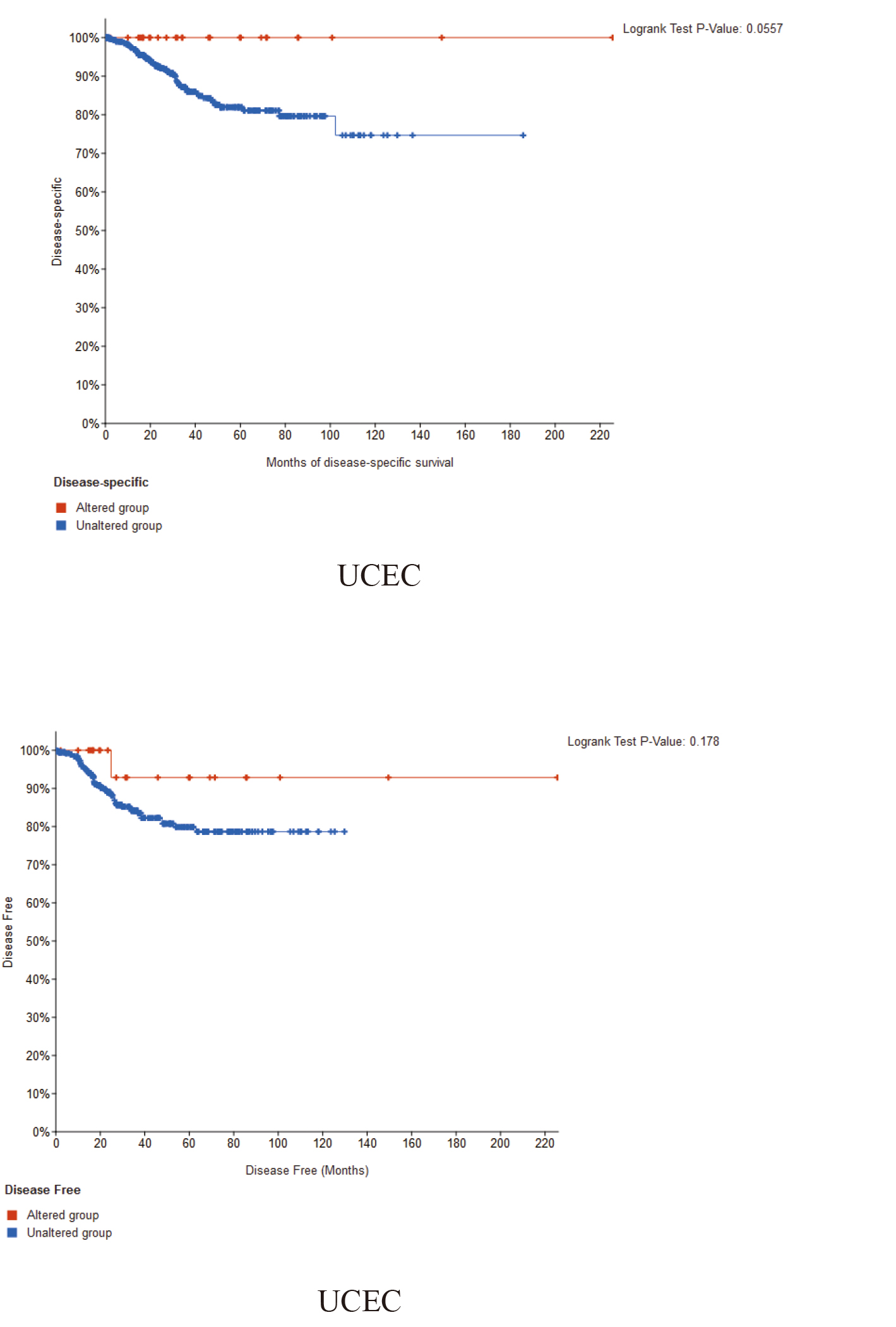

Supplement: Supplementary file 2 [file Image3.JPEG]

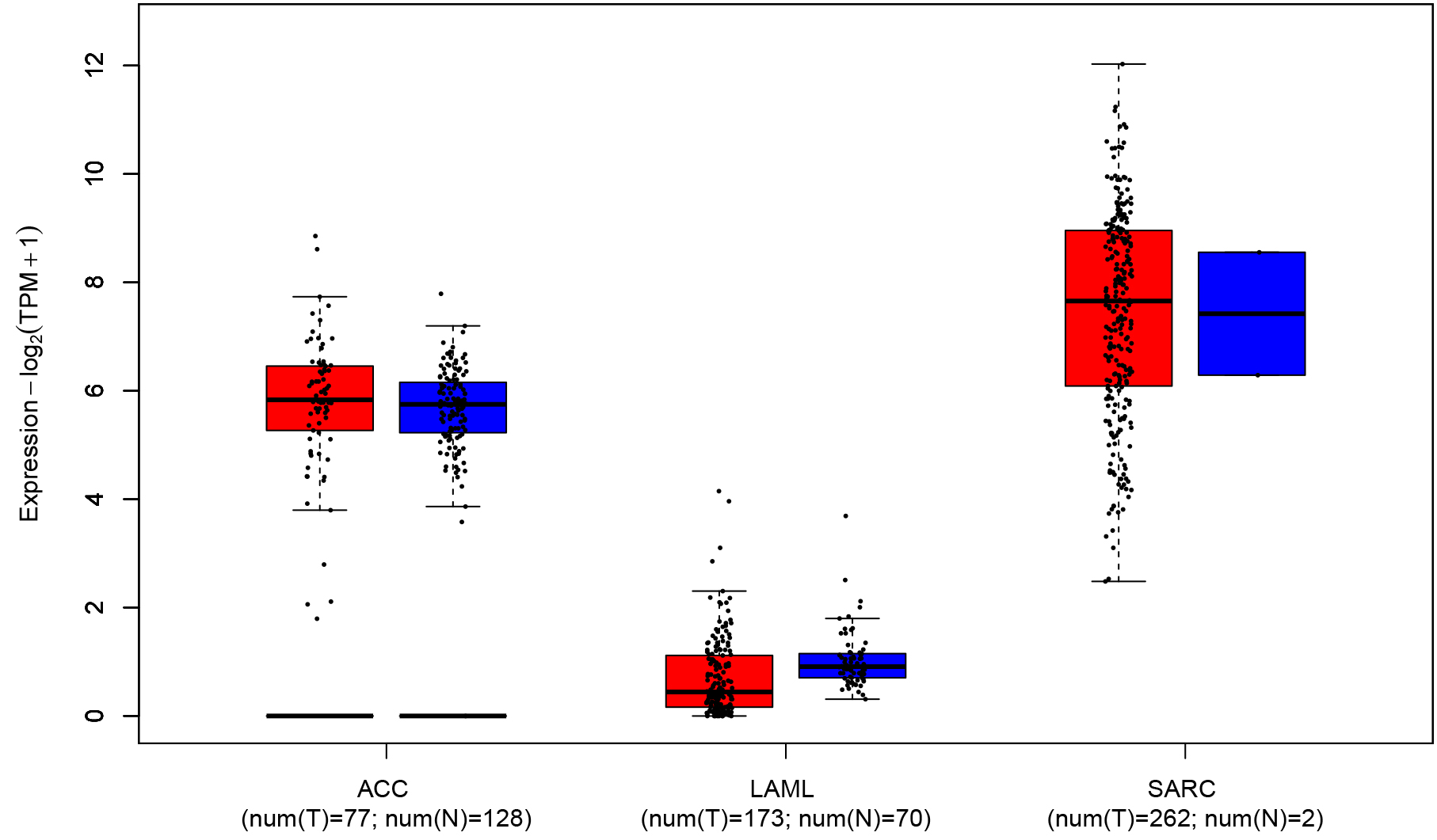

Supplement: Supplementary file 5 [file Image1.JPEG]

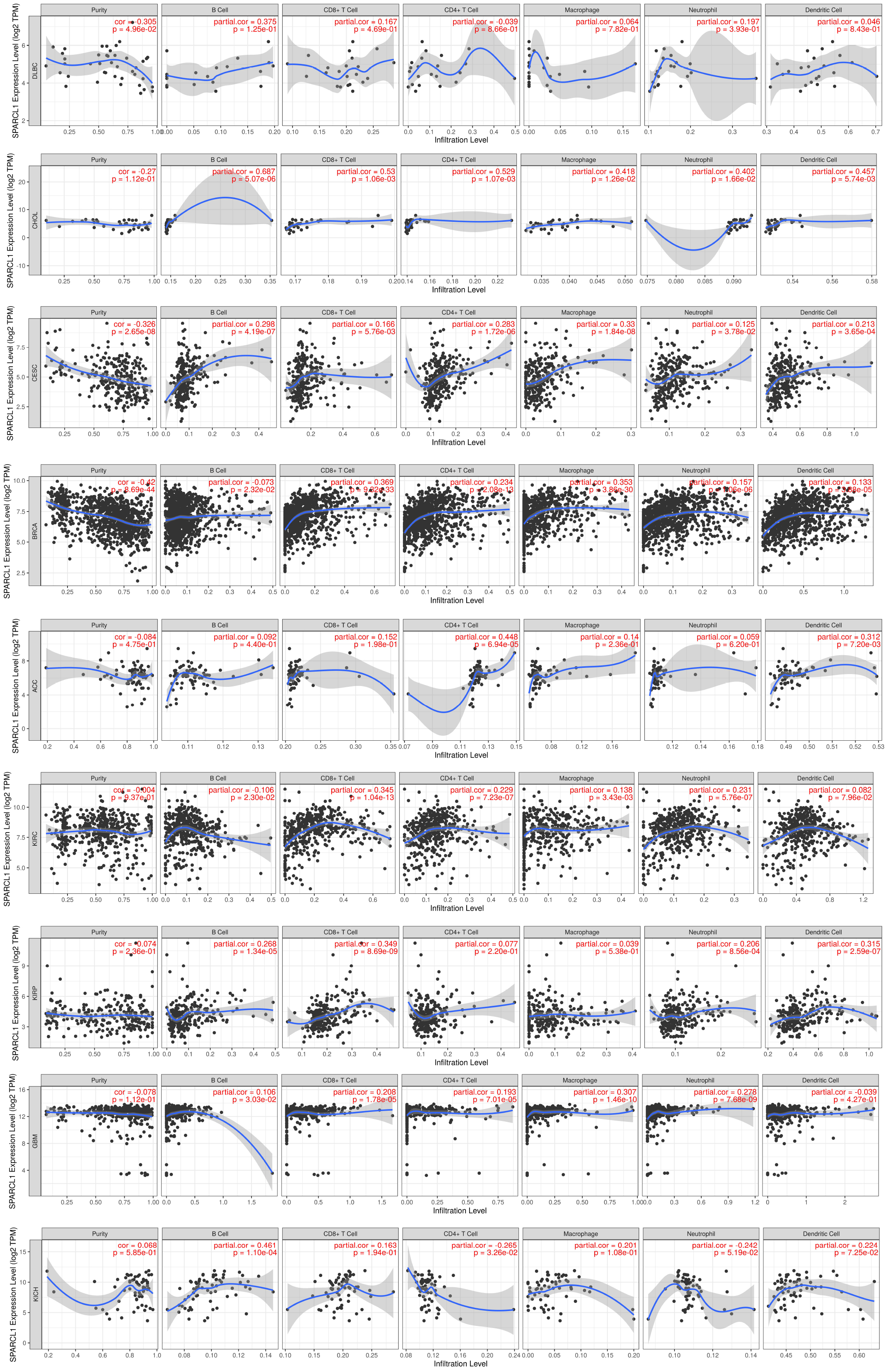

Supplement: Supplementary file 6 [file Image4.JPEG]

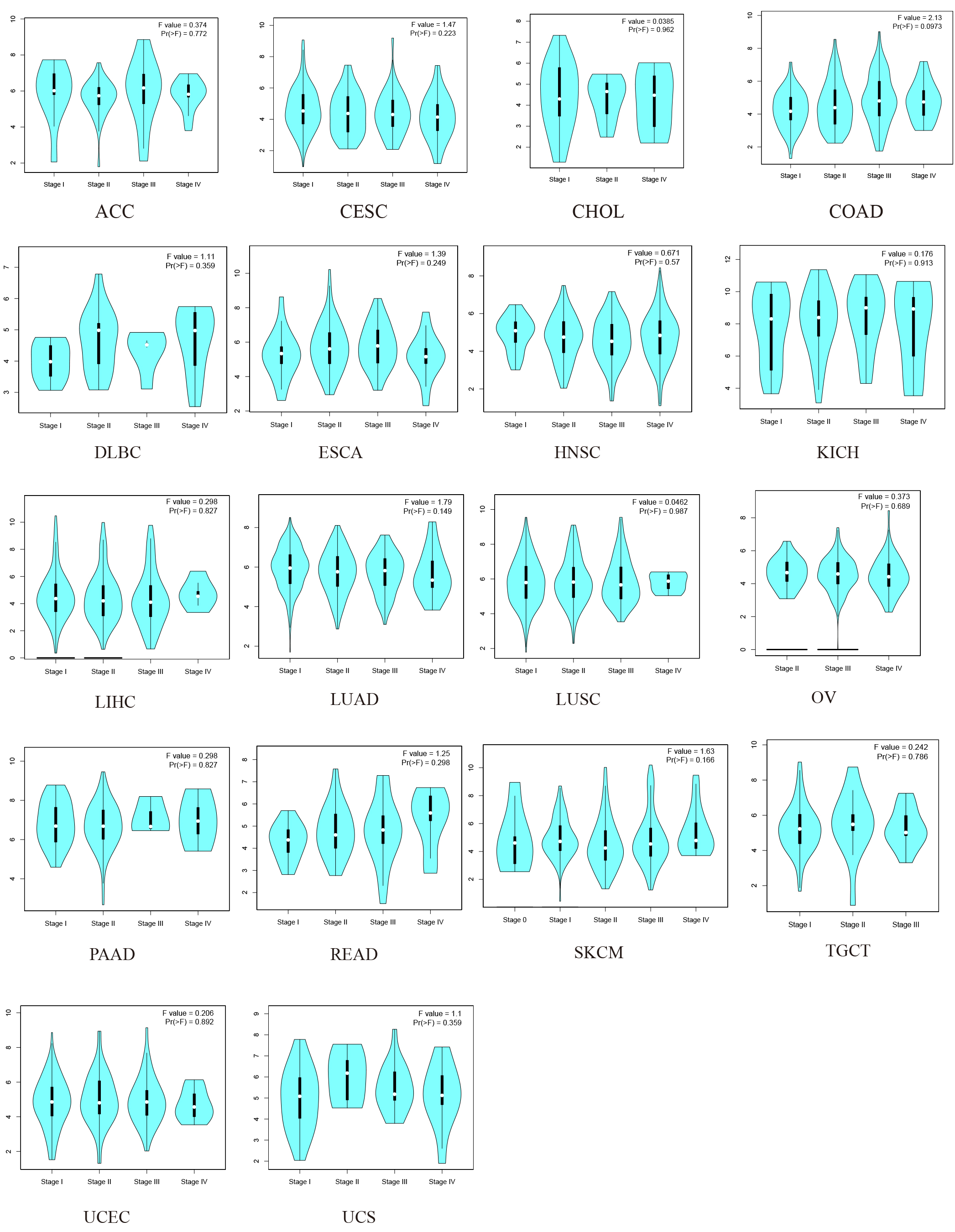

Supplement: Supplementary file 7 [file Image2.JPEG]

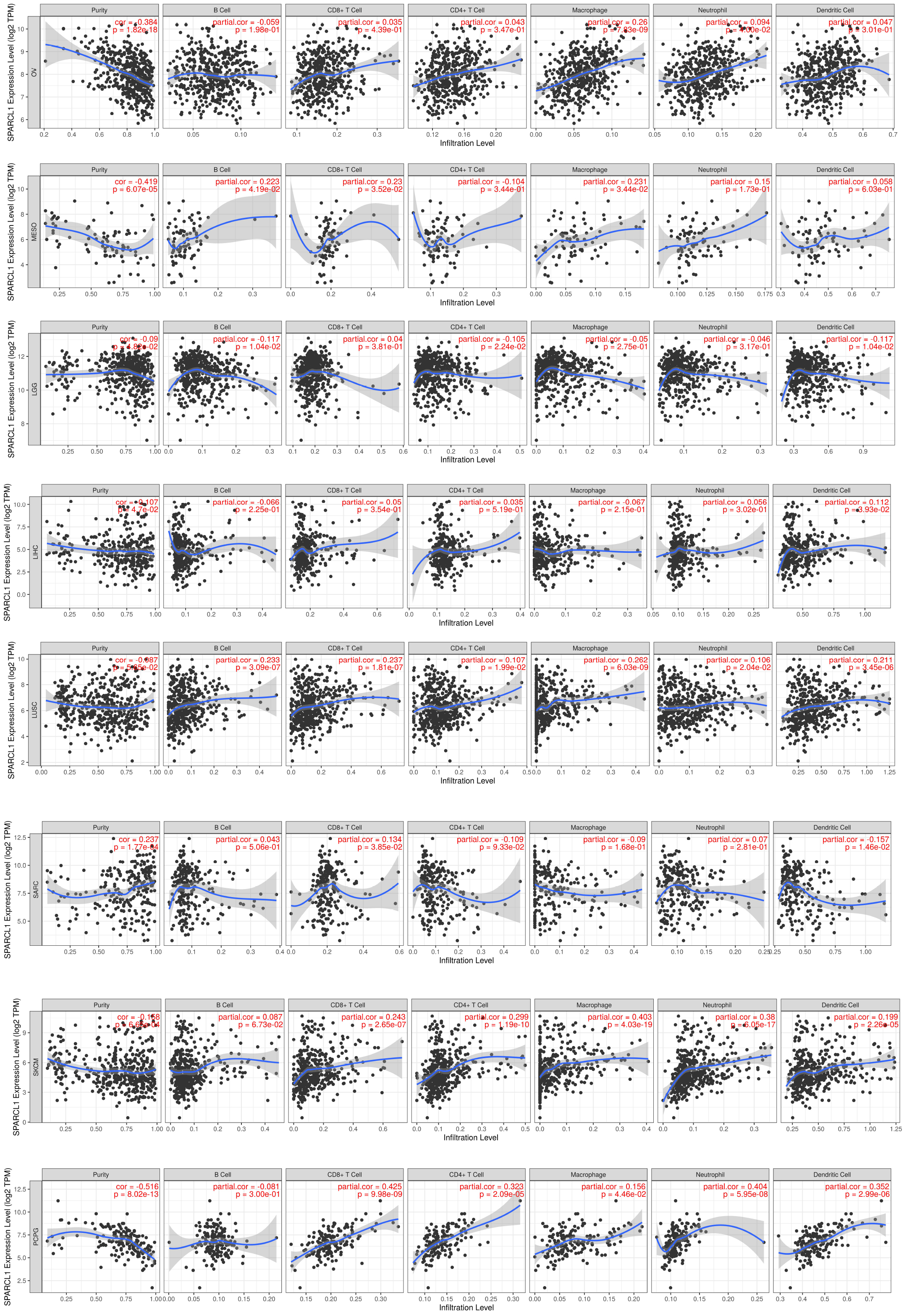

Supplement: Supplementary file 8 [file Image5.JPEG]

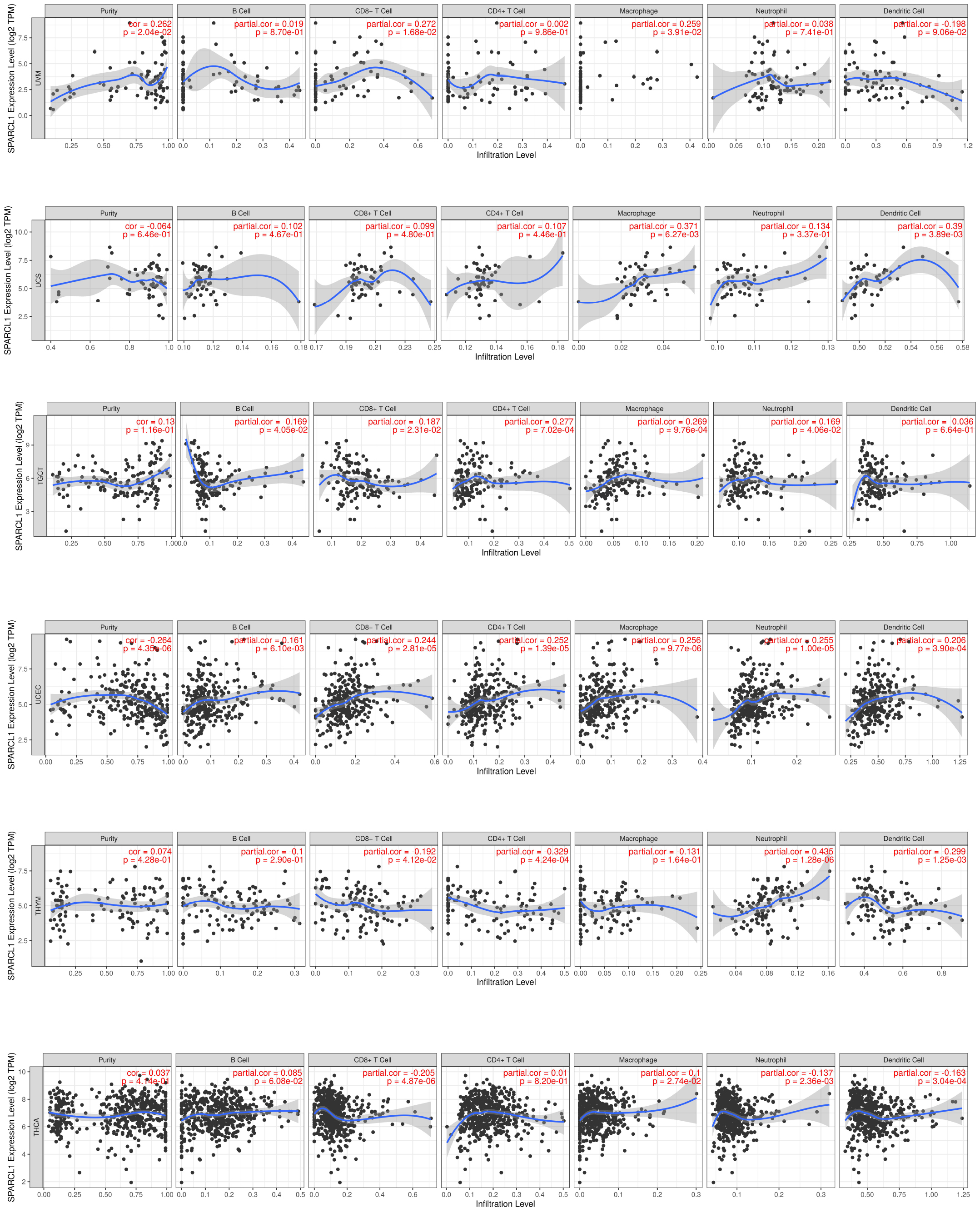

Supplement: Supplementary file 12 [file Image6.JPEG]
