# Supplementary material for: Immunological role and prognostic value of SPARCL1 in pan-cancer analysis
Source: Pathol Oncol Res. 2022 Nov 22;28:1610687. doi: 10.3389/pore.2022.1610687 (PMC9722748; doi:10.3389/pore.2022.1610687)
Supplement: Supplementary file 10 [file DataSheet2.DOCX]

library("clusterProfiler")

library("org.Hs.eg.db")

library("enrichplot")

library("ggplot2")

pvalueFilter=0.05

qvalueFilter=1

rt=read.table("id.txt",sep="\t",header=T,check.names=F)

gene=rt$entrezID

colorSel="qvalue"

if(qvalueFilter>0.05){

colorSel="pvalue"

}

kk <- enrichKEGG(gene = gene, organism = "hsa", pvalueCutoff =1, qvalueCutoff =1)

KEGG=as.data.frame(kk)

KEGG$geneID=as.character(sapply(KEGG$geneID,function(x)paste(rt$gene[match(strsplit(x,"/")[[1]],as.character(rt$entrezID))],collapse="/")))

KEGG=KEGG[(KEGG$pvalue<pvalueFilter & KEGG$qvalue<qvalueFilter),]

write.table(KEGG,file="KEGG.txt",sep="\t",quote=F,row.names = F)

pdf(file="KEGGresults.pdf",width = 10,height = 7)

barplot(kk, drop = TRUE, showCategory = 30, color = colorSel)

dev.off()
