# Supplementary material for: Immunological role and prognostic value of SPARCL1 in pan-cancer analysis
Source: Pathol Oncol Res. 2022 Nov 22;28:1610687. doi: 10.3389/pore.2022.1610687 (PMC9722748; doi:10.3389/pore.2022.1610687)
Supplement: Supplementary file 11 [file DataSheet1.DOCX]

Supplement Fig. 1 The expression of SPARCL1 in ACC, LAML, and SARC.

Supplement Fig. 2 The expression level of SPARCL1 was not associated with the pathological stages of ACC, CESC, CHOL, COAD, DLBC, ESCA, HNSC, KICH, LIHC, LUAD, LUSC, OV, PAAD, READ, SKCM, TGCT, UCEC, and USC.

Supplement Fig. 3 UCEC patients with altered SPARCL1 had not a better DFS and DSS than those with unaltered SPARCL1.

Supplement Fig. 4-6 Correlation of SPARCL1 with immune infiltration level in other cancer types.
